# Regional versus General Anesthesia in Postoperative Pain Management after Distal Radius Fracture Surgery: Meta-Analysis of Randomized Controlled Trials

**DOI:** 10.3390/jpm13111543

**Published:** 2023-10-27

**Authors:** Young Hak Roh, Seong Gyun Park, Seung Hyun Lee

**Affiliations:** Department of Orthopaedic Surgery, Ewha Womans University Medical Center, Ewha Womans University College of Medicine, 1071 Anyangcheon-ro, Yangcheon-gu, Seoul 07985, Republic of Korea; silbsk15@naver.com (S.G.P.); priestman@naver.com (S.H.L.)

**Keywords:** distal radius fracture, regional anesthesia, general anesthesia, postoperative pain, meta-analysis, randomized controlled trials

## Abstract

Distal radius fractures are the most prevalent upper extremity fractures, posing a significant public health concern. Recent studies comparing regional and general anesthesia for postoperative pain management after these fractures have yielded conflicting results. This meta-analysis aimed to compare the effectiveness of regional and general anesthesia concerning postoperative pain management and opioid consumption following distal radius fracture surgery. A comprehensive search was conducted in PubMed, Cochrane Library, and EMBASE databases to identify relevant randomized controlled trials. Four randomized trials involving 248 participants were included in the analysis. A pooled analysis revealed that regional anesthesia led to significantly reduced postoperative pain scores at 2 h compared to general anesthesia (SMD −2.03; 95% CI −2.88–−1.17). However, no significant differences in pain scores were observed between the two anesthesia types after 12 h post-surgery. Regional anesthesia was associated with lower total opioid consumption (SMD −0.76; 95% CI −1.25–−0.26) and fewer occurrences of nausea and vomiting compared to the general anesthesia. Nonetheless, opioid consumption on the first day post-discharge was significantly higher in the regional anesthesia group (SMD 0.83; 95% CI 0.47–1.20). The analgesic superiority of regional anesthesia is confined to the early postoperative hours with overall lower opioid use but a notable increase in opioid consumption on the first day post-discharge, potentially attributable to rebound pain.

## 1. Introduction

Distal radius fractures are the most frequently encountered fractures in the upper extremities and pose a significant public health concern, especially among the elderly [1]. Numerous studies have documented the rising prevalence of distal radius fractures as the active elderly population continues to grow [2]. These fractures make a substantial contribution to the overall morbidity in the elderly population and impose a significant socioeconomic burden [3]. Surgical fixation of distal radius fractures allows early initiation of motion exercises after surgery, leading to a faster resumption of daily activities than nonsurgical treatment methods [4,5]. However, postoperative pain can act as a deterrent to the early initiation of exercise, potentially reducing patient satisfaction and leading to suboptimal clinical outcomes owing to delayed rehabilitation [6].

Surgical fixation of distal radial fractures can be performed under general (GA) or regional anesthesia (RA). Regional anesthetic nerve blocks have a positive impact on various aspects of the surgical process, such as reduced acute postoperative pain, decreased opioid consumption, and improved patient satisfaction [7]. However, several studies have emphasized the considerable risk of “rebound pain” once the effects of RA wear off, with an incidence rate of approximately 40–50% reported following peripheral nerve block procedures [8,9,10]. A previous comprehensive meta-analysis investigating the use of various types of anesthesia for the treatment of distal radius fractures did not find substantial evidence favoring any particular method [11].

Randomized controlled trials comparing RA with GA for postoperative pain management after distal radius fractures have reported conflicting findings. Two early randomized controlled trials [12,13] reported no overall differences in analgesia after distal radial fracture fixation. Both studies found that RA was associated with rebound pain and worse pain scores than GA at approximately 12 h after surgery. However, more recently published randomized trials [14,15] reported contradictory results, with some indicating that RA provides better pain relief and higher patient satisfaction than GA for distal radius fracture fixation.

Although some reviews have been published on this topic, the studies included mixed levels of evidence and lacked quantitative analysis [11,16]. Therefore, we conducted a meta-analysis with evidence based solely on randomized controlled trials. This meta-analysis aimed to identify the effects of anesthesia on postoperative pain, opioid consumption, and occurrence of nausea and vomiting after surgery for distal radius fracture.

## 2. Materials and Methods

### 2.1. Study Strategy

This meta-analysis was conducted according to the Preferred Reporting Items for Systematic Review and Meta-Analysis (PRISMA) statement [17]. We did not register or publish a prior protocol of this study (see Supplementary Material). Search was conducted between April 2023 and July 2023.

Pubmed, Cochrane Central Register of Controlled Trials, and EMBASE database were searched for articles with language restricted to English. Initially, we did not restrict the research type or year of publication to avoid missing relevant articles. Search algorithms were developed for each database (without a set time limit). A combination of search terms was employed, encompassing the title, abstract, Medical Subject Headings (MeSH), Emtree, and keywords. The search terms included “wrist fracture[MeSH]”, “distal radius fracture[tiab]”, “anesthesia[MeSH]”, “regional anesthesia[tiab]”, “nerve block[tiab]”, “brachial plexus[tiab]” and “general anesthesia[tiab].

### 2.2. Study Selection

Two authors (YHR, SGP) independently conducted the initial database search and screened the titles and abstracts for eligibility. The full manuscript was then reviewed for inclusion and exclusion criteria. Discrepancies were resolved by consensus among the reviewers. We also performed manual searches of the references in the relevant articles and previous systemic reviews to avoid missing eligible articles.

We used the Population, Intervention, Comparator, Outcome and Study design (PICOS) framework to formulate eligibility criteria [17]. Studies were included if they met the following selection criteria: participants, patients with distal radius fractures treated surgically; intervention, regional anesthesia; comparison, general anesthesia; outcome, postoperative pain; and design, randomized controlled trials. The exclusion criteria were mixed patient populations with indistinguishable datasets, publications that did not provide concrete data, patient populations under 18 years of age, and papers written in a language other than English.

### 2.3. Quality Assessment

The methodological quality of the studies was evaluated using the Cochrane Collaboration’s Risk of Bias Tools [18]. These criteria were used to assess the potential bias level for each of the following components: random sequence generation (selection bias), allocation concealment (selection bias), double-blinding (performance or detection bias), selective data reporting (reporting bias), and missing data (attrition bias). Two reviewers (YHR and SGP) independently assessed the methodological quality of the studies and unresolved disagreements were resolved by consensus through consultation with a third reviewer.

### 2.4. Data Extraction and Outcome Measure

Data extraction and outcome measurement were conducted independently by two authors (SGP and SHL). Any discrepancies that emerged were subject to discussion, and final decisions were reached through mutual agreement among the evaluators. We employed a pre-designed data spreadsheet for data extraction. It included key information such as author names, publication year, study design, sample size, patient demographics (age, sex, affected side, etc.), anesthesia methods, primary outcomes, and complications.

The following outcomes were investigated: postoperative pain levels, opioid consumption (in equivalent units), and occurrences of nausea and vomiting.

### 2.5. Statistical Analysis

The primary outcomes of this meta-analysis included postoperative pain levels and opioid consumption, with the secondary outcome being the incidence of nausea and vomiting following distal radius fracture surgery. We conducted a pooled analysis to compare multiple clinical outcome measures between the groups depending on the availability of the data. For all comparisons, standardized mean differences (SMD) with 95% confidence intervals (CI) were used for continuous variables, while odds ratios (OR) with 95% CI were calculated for binary variables. When medians with interquartile ranges were reported without means with standard deviations (SD), SDs were calculated using the quantile estimation approach [19]. Between-study heterogeneity was determined using the I^2^ statistics. Values > 25%, >50%, and >70% were regarded as low, moderate, and high heterogeneity, respectively. Random effects models for meta-analysis were applied in the presence of moderate to high heterogeneity (I^2^ > 50%); otherwise, fixed effects models were used. The inverse variance weighting method was employed to derive pooled estimates of SMD or OR, along with their respective 95% CI. The results of the meta-analysis were considered statistically significant at *p* < 0.05. Subgroup analysis was only conducted for postoperative VAS pain at 24 h to explore potential explanations for the observed heterogeneity, given the limited number of articles available. A sensitivity analysis was conducted by omitting single eligible studies to evaluate the influence of each study in primary outcomes. A funnel plot was originally planned to detect publication bias but was excluded from the analysis owing to its limited interpretability caused by the small number of included studies. Statistical analyses were performed using the Comprehensive Meta-analysis Software version 3 (Biostat, Englewood, NJ, USA) and Review Manager (version 5.3; Cochrane Collaboration, Copenhagen, Denmark).

## 3. Results

### 3.1. Characteristics and Methodological Quality of the Included Study

**Figure 1:**
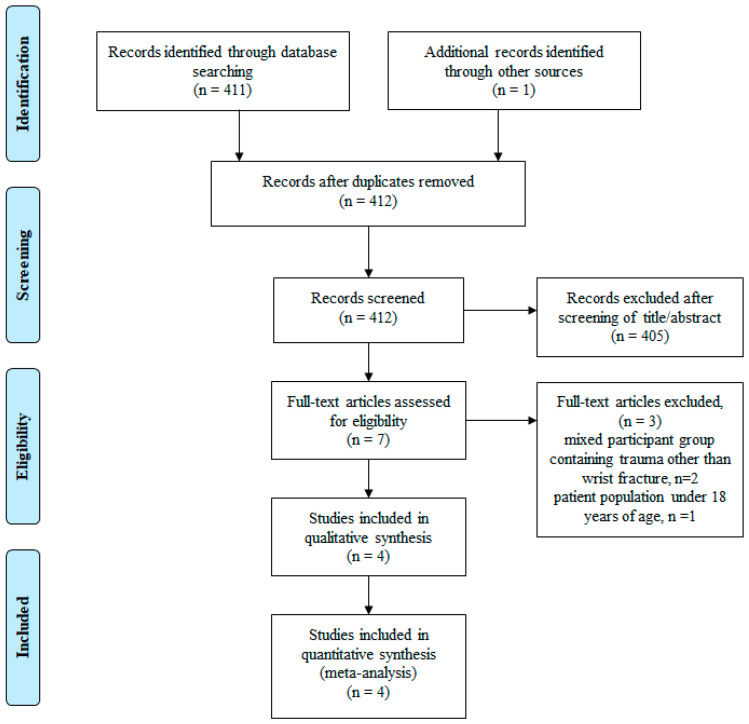
Preferred Reporting Items for Systematic Review and Meta-Analysis (PRISMA) flow diagram of literature selection.

The initial search yielded 412 articles, 4 of which met the inclusion and exclusion criteria and underwent qualitative analysis (Figure 1). All four studies included in this analysis were randomized controlled trials published in English between 2016 and 2022. Table 1 summarizes the key characteristics of these studies. Among them, two studies utilized the infraclavicular block approach, whereas one study employed the supraclavicular block approach and another study used the axillary block approach. The duration of acute postoperative pain assessment varied from 24 to 72 h across the studies.

**Figure 2:**
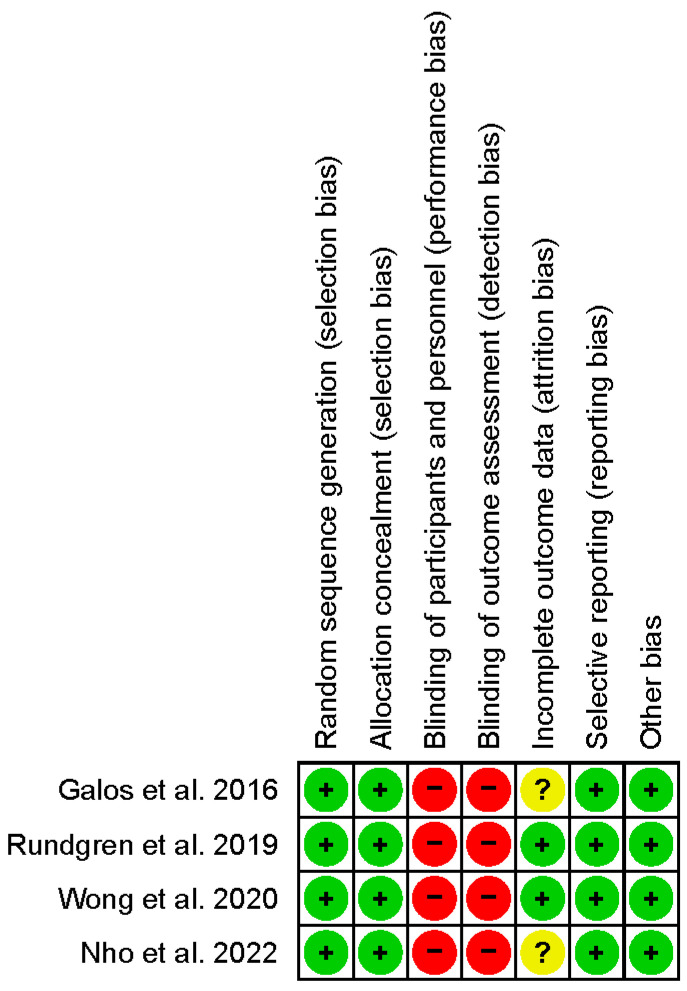
Methodological quality of the included randomized controlled trials. The risks of bias items were assessed and categorized as “low risk” (+), “unclear risk” (?), or “high risk” (−). Galos et al., 2016 [12]; Rundgren et al., 2019 [13]; Wong et al., 2020 [14]; Nho et al., 2022 [15].

Figure 2 summarizes the risk-of-bias results of the studies. Except for the absence of blinding of patients regarding the anesthesia technique and the absence of blinding of the research team responsible for collecting postoperative outcome data, the overall methodological quality of the studies was acceptable.

### 3.2. Outcome

#### 3.2.1. Postoperative VAS Pain Score at 2, 12, 24, and 48 h

**Figure 3:**
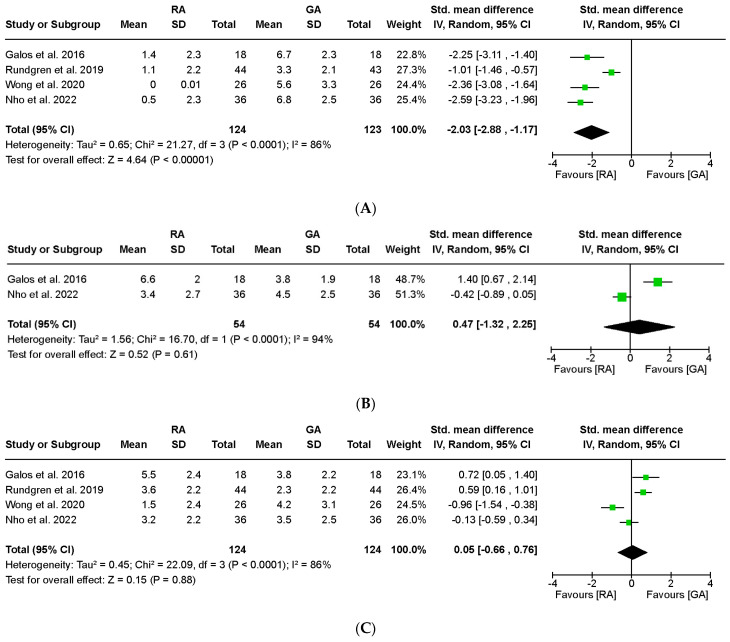

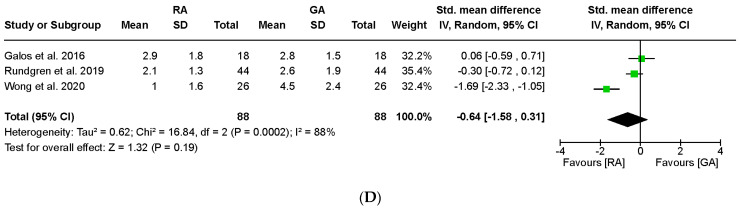
Forest plot of comparison in acute postoperative pain after regional or general anesthesia for distal radius fracture surgeries: (**A**) at 2 h, (**B**) at 12 h, (**C**) at 24 h, and (**D**) at 48 h. RA = regional anesthesia, GA = general anesthesia, SD = standard deviation, IV = inverse variance, CI = confidence interval. Galos et al., 2016 [12]; Rundgren et al., 2019 [13]; Wong et al., 2020 [14]; Nho et al., 2022 [15].

The postoperative pain score through visual analogue scale (VAS) at 2 h was assessed in the four studies, which involved 247 wrists. All four studies consistently showed that the RA group had lower pain scores than the GA group at 2 h. The meta-analysis demonstrated significantly lower VAS scores in the RA group than the GA group. The standardized mean difference (SMD) was calculated as −2.03, with a 95% confidence interval (CI) ranging from −2.88 to −1.17 (Figure 3A). The results of the sensitivity analysis did not differ from those of the original studies.

Two studies involving 108 wrists examined VAS pain scores at 12 h. One study demonstrated significantly higher pain VAS scores in the RA group than the GA group at 12 h, whereas another study found no significant difference in pain scores between the groups. A meta-analysis revealed that the pooled effect size was not significant (Figure 3B; SMD 0.47; 95% CI −1.32–2.25; I^2^ = 94%).

The VAS pain score at 24 h was assessed in four studies involving 248 wrists. Two studies reported higher VAS pain scores in the RA group compared than in the GA group, whereas one study reported higher pain scores in the GA group. The results of the meta-analysis indicated that the pooled effect size was not significant with high heterogeneity (Figure 3C; SMD 0.05; CI at 95% −0.66–0.76; I^2^ = 86%). The heterogeneity was resolved when the investigations were pooled within the subgroup on outpatient surgical settings (Table 2). The sensitivity analysis did not reveal any significant differences in the outcome.

The pain score at 48 h was assessed in three studies with a total of 176 wrists. One study reported higher pain VAS scores in the GA group than in the RA group, whereas the other two investigations did not find any significant differences between the groups. The results of the meta-analysis showed no significant difference in the VAS pain scores between the two groups at 48 h (Figure 3D; SMD −0.64; CI at 95% −1.58–0.31; I^2^ = 88%). The sensitivity analysis revealed no change in the result of the original analysis.

#### 3.2.2. Amount of Opioid Consumption

**Figure 4:**
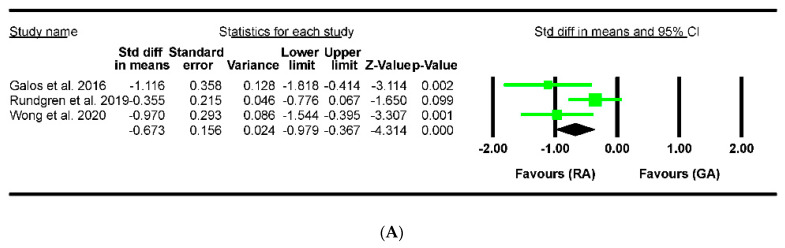

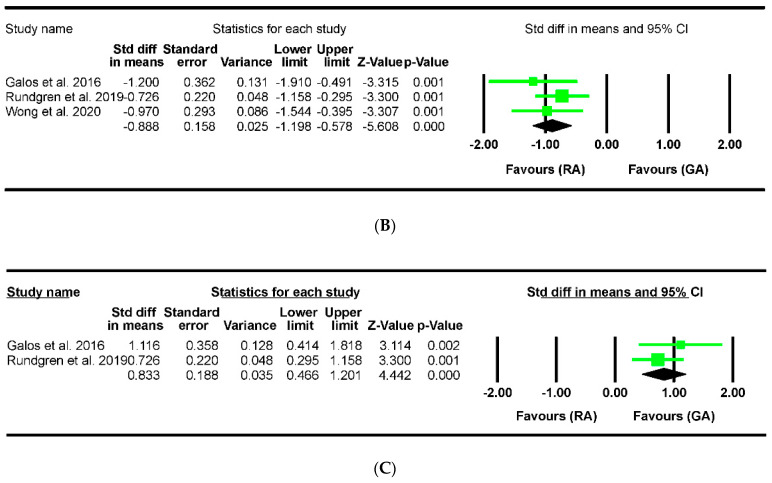
Forest plot of comparison in postoperative opioid consumption after regional or general anesthesia for distal radius fracture surgeries: (**A**) total, (**B**) in PACU, and (**C**) on the first day after discharge. RA = regional anesthesia, GA = general anesthesia, CI = confidence interval. Galos et al., 2016 [12]; Rundgren et al., 2019 [13]; Wong et al., 2020 [14].

The total amount of opioid consumption after distal radius fracture surgery was reported in three studies that included 132 wrists. Among these studies, two reported a higher total opioid consumption in the GA group than in the RA group, whereas the remaining study did not find a significant difference in opioid consumption between the two groups. The results of the meta-analysis demonstrated that the GA group had significantly greater total opioid consumption than the RA group (Figure 4A, SMD −0.76; CI at 95% −1.25–−0.26; I^2^ = 58%).

Three studies consistently reported higher opioid consumption in the post-anesthesia care unit (PACU) or before discharge in the GA group than in the RA group. The meta-analysis indicated a significant increase in opioid consumption in the RA group compared to the GA group during this period (Figure 4B, SMD −0.89; CI at 95% −1.12–−0.58; I^2^ = 0%).

Opioid consumption after postoperative discharge, specifically on the first day, was evaluated in two investigations focusing on ambulatory surgery. Both studies reported higher opioid use in the RA group than in the GA group. The results of the meta-analysis indicated that the patients in the RA group had significantly greater opioid use than those in the GA group (Figure 4C, SMD 0.83; CI at 95% 0.47–1.20; I^2^ = 0%).

#### 3.2.3. Postoperative Nausea and Vomiting

**Figure 5:**
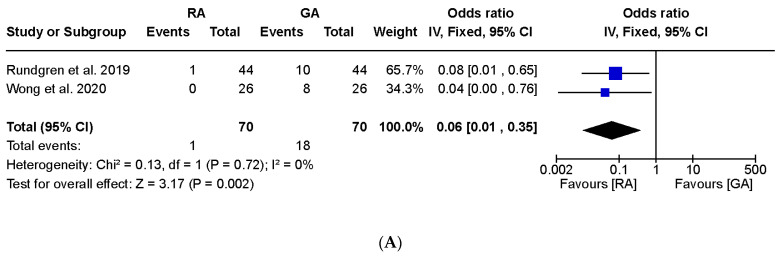

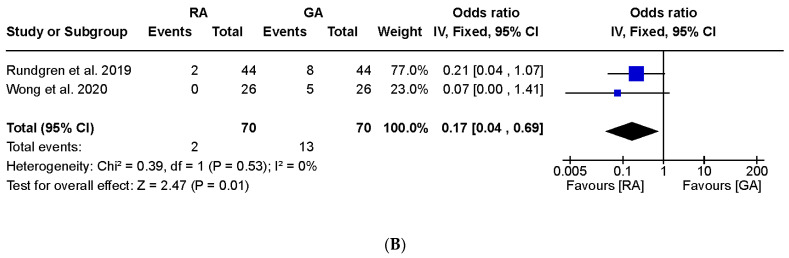
Forest plot of comparison in postoperative nausea and vomiting after regional or general anesthesia for distal radius fracture surgeries: (**A**) nausea and (**B**) vomiting. RA = regional anesthesia, GA = general anesthesia, SD = standard deviation, SD = standard deviation, IV = inverse variance, CI = confidence interval. Rundgren et al., 2019 [13]; Wong et al., 2020 [14].

Postoperative nausea and vomiting were assessed in two studies involving 140 wrists. Both studies consistently revealed a higher incidence of postoperative nausea and vomiting in the GA group than in the RA group. The results of the meta-analysis indicated a lower risk of postoperative nausea (Figure 5A; OR, 0.06, 95% CI, 0.01 to 0.35, I^2^ = 0%) and vomiting (Figure 5B; OR, 0.17, 95% CI, 0.04 to 0.69, I^2^ = 0%) in patients who underwent RA compared to those who received GA.

## 4. Discussion

Prior investigations comparing RA and GA for postoperative pain management have yielded conflicting results, posing a significant challenge to clinicians in deciding which mode of anesthesia to employ in order to enhance post-operative recovery and reduce oral opioid use. In this meta-analysis, we identified four randomized controlled trials that directly compared RA and GA for postoperative pain management after distal radius fracture surgery. This analysis aimed to provide insights into the optimal choice of anesthesia for managing postoperative pain in this specific surgical context. Furthermore, it provides insight into rebound pain following RA, an aspect often overlooked in the context of distal radius fracture fixation. Our meta-analysis revealed significant differences in the early postoperative pain and opioid consumption based on the anesthesia method. Specifically, the GA group had significantly higher pain scores at 2 h compared to the RA group; however, opioid consumption after postoperative discharge on the first day was significantly increased in the RA group compared to in the GA group. The total opioid consumption was greater in the GA group than in the RA group. However, no significant differences were observed between the two groups in the VAS pain scores after 12 h.

RA was associated with decreased postoperative pain scores at 2 h compared to GA. However, the association diminished after the early postoperative period, and no significant differences in pain scores were observed between the GA and RA group after 12 h. The early postoperative pain patterns were significantly influenced by the anesthesia method. Patients with RA consistently exhibited a rapid increase in pain scores on the first operative day once the effects of regional anesthesia wore off. Some patients experienced an abrupt increase in pain, necessitating more pain medications. In contrast, patients with GA showed a continuous decrease in pain during the early postoperative period. Patients who received RA for ambulatory wrist fracture surgery reported a higher rate of unplanned healthcare resource utilization caused by post-discharge pain than those undergoing GA [20]. The heterogeneity in pain scores within the RA group between 12 and 24 h may have been influenced by the incidence or extent of rebound pain. Interestingly, two studies [12,13] that reported high pain scores at 12 or 24 h were conducted in outpatient surgery settings, whereas investigations [14,15] that did not show higher pain scores during this time were conducted in inpatient surgery settings. It is likely that rebound pain was more efficiently controlled in inpatient surgical settings, as previously demonstrated by the lower opioid consumption rates in shoulder surgeries performed in inpatient surgical settings than in those performed in outpatient surgical settings [21]. Patients with distal radius fractures are frequently admitted to hospital for a few days in some Asian countries, where admission costs are relatively low and patients tend to have longer hospital stays [6]. The management of rebound pain, especially in ambulatory surgical settings, is an important consideration.

Opioids continue to have a significant role for postoperative pain management, but their use in the postoperative period may elevate the risk of adverse effects, including nausea and vomiting, respiratory depression, sedation, pruritus, urinary retention, and sleep disturbances [6]. Total opioid consumption was lower in the RA group, and there were fewer instances of nausea and vomiting compared to the GA group. However, opioid consumption on the first day after discharge was significantly higher in the RA group than that in the GA group. As previously mentioned, this phenomenon can be attributed to rebound pain [8]. Rebound pain has been previously demonstrated in various upper extremity surgeries [6,22], although not reported in every investigation [14]. The results are consistent with previous findings showing that RA reduces opioid demand during the inpatient stage, but not during the outpatient period following extremity fracture surgeries [23,24]. Controversy also exists regarding the total opioid consumption after GA and RA in fracture surgeries. Several recent retrospective investigations specifically examining upper- and lower-extremity fractures have shown that RA was associated with increased inpatient and outpatient opioid demands after adjusting for baseline patient and treatment characteristics [22,25,26]. These findings contradict traditional expectations regarding the opioid-sparing effects of RA [27,28]. The observed heterogeneity may have resulted from various factors such as different types of injuries and surgeries, variation in RA technique, adjuvant medications used, and perioperative multimodal analgesia techniques employed. These factors may influence the incidence and severity of rebound pain, consequently affecting opioid consumption after RA for fracture surgeries.

The higher incidence of nausea and vomiting in the GA group could be attributed to the differences in total opioid consumption. Postoperative nausea and vomiting are known to be influenced by various factors associated with the patient, surgery, and anesthesia [29]. Previous studies demonstrated that postoperative nausea and vomiting are strongly influenced by postoperative opioid use in a dose-dependent manner [30]. This effect appears to persist as long as opioids are used for pain management in the postoperative period [31]. A substantial number of patients undergoing ambulatory hand surgery experience distressing levels of postoperative nausea and vomiting, and dissatisfaction with ambulatory hand surgery correlated with moderate as well as high levels of postoperative nausea and vomiting [32].

The main limitation of this meta-analysis is the small number of included studies. The inclusion criteria were limited to randomized controlled trials, resulting in a limited pool of studies eligible for the meta-analysis. However, it was chosen to ensure a high level of evidence and facilitate high-grade recommendations. Additionally, heterogeneity in outcome reporting was observed among the included studies. Not all of the included studies provided data on opioid consumption, and the timing of follow-up assessments varied across studies. Further research with larger sample sizes and diverse study settings is warranted to provide a more comprehensive understanding of this topic. In addition, treatment-related variables, including the time to surgery and initial closed reductions before surgery, and injury characteristics, such as open or closed fractures, could potentially influence postoperative pain level after distal radius fracture [33,34], but these potentially confounding variables were not addressed in this meta-analysis due to limitations in the available information from the included studies. Another limitation of this meta-analysis was the variability in the postoperative pain management protocols among the included trials. Although these variations reflect actual clinical practice (real-world settings), these introduced a potential source of heterogeneity into the analysis. To compensate for this inherent variability, our study examined postoperative opioid consumption and the incidence of nausea and vomiting as additional outcome measures.

## 5. Conclusions

The use of RA for distal radial fracture surgery was associated with reduced postoperative pain scores during the early postoperative period only. However, after 12 h, no significant differences in the VAS pain scores were observed between the GA and RA groups. Total opioid consumption was higher in the GA group than in the RA group. However, opioid consumption on the first day after discharge was significantly higher in the RA group, potentially because of rebound pain. These findings provide valuable insights for the selection of an appropriate anesthetic method based on patient characteristics and specific trauma surgeries.

## Figures and Tables

**Table 1:** Characteristics of the included studies. RA = regional anesthesia, GA = general anesthesia, RCT = randomized controlled trial, AO/OTA = Arbeitsgemeinschaft für Osteosynthesefragen/Orthopaedic Trauma Association.

Study	Study Design	Sample Size		Mean Age (yr)		Female (%)		Injury to Right/Dominant Hand (%)		AO/OTA Fracture Type	Postoperative Pain Evaluations
		RA	GA	RA	GA	RA	GA	RA	GA		
Galos et al. (2016) [12]	RCT	18	18	54.4	54.9	50	66.6	Not reported		Type A, *n* = 9 Type B, *n* = 2 Type C, *n* = 23	2, 4, 6, 12, 24, 48, and 72 h
Rundgren et al. (2019) [13]	RCT	44	44	51.2	53.2	75	77	34	43	Type A, *n* = 35 Type B, *n* = 15 Type C, *n* = 38	0, 2, 24, 48, and 72 h, 2 weeks, and 6 months
Wong et al. (2020) [14]	RCT	26	26	59.2	58.9	69.2	73.1	42.3	42.3	Not reported	0, 1, 2, 24, and 48 h, 3 and 6 moths
Nho et al. (2022) [15]	RCT	36	36	69.5	71.2	100	100	61.1	38.9	Type A, *n* = 4 Type B, *n* = 0 Type C, *n* = 68	2, 4, 6, 12, and 24 h

**Table 2:** Results of subgroup analysis for postoperative pain at 24 h according to surgical settings for regional anesthesia.

Subgroup	No. Studies	SMD; 95% CI	Heterogeneity
Outpatient surgical settings	2	0.65; 0.23–1.06	I^2^ = 0%, *p* = 0.74
Inpatient surgical setting	2	−0.53; −1.36–0.30	I^2^ = 80%, *p* = 0.025
RA with infraclavicular approach	2	−0.13; −1.8–1.6	I^2^ = 93% *p* = 0.01
RA with supraclavicular approach	1	0.59; 0.07–1.11	NA
RA with axillary approach	1	−0.13; −0.59–0.34	NA

RA = regional anesthesia, SMD = standardized mean differences, CI = confidence intervals, NA = not available.

## Data Availability

The data presented in this study are available upon request to the corresponding author.

## Supplementary Materials

The following supporting information can be downloaded at: https://www.mdpi.com/article/10.3390/jpm13111543/s1.
